# Alzheimer's disease: A comprehensive review of epidemiology, pathophysiology, diagnosis, and treatment

**DOI:** 10.3934/Neuroscience.2026009

**Published:** 2026-04-10

**Authors:** Arosh S. Perera Molligoda Arachchige, Bianca Schmiliver, Gabriel Amorim Moreira Alves

**Affiliations:** 1 University of Milan, Via Festa del Perdono 7, 20122 Milan, Italy; 2 Humanitas University, Via Rita Levi Montalcini 4, 20072 Pieve Emanuele, Italy

**Keywords:** Alzheimer's disease, treatments, pathophysiology, dementia, diagnosis, imaging

## Abstract

Alzheimer's disease (AD) is the most common cause of dementia, representing a major global public health challenge as populations age. It accounts for roughly 60%–80% of all dementia and is characterized by progressive cognitive decline, memory impairment, and eventual loss of independence in daily functioning. The disease unfolds over decades, with neuropathological alterations preceding the onset of clinical symptoms by many years. Alzheimer's disease is closely linked to the accumulation and deposition of cerebral amyloid-β (Aβ) and represents the most common cerebral amyloid deposition disorder. Recent advances in molecular biology, neuroimaging, and biomarker science have revealed a complex, multifactorial pathogenesis involving protein misfolding, neuroinflammation, synaptic dysfunction, vascular factors, and network-level propagation of pathology. This review synthesizes current knowledge on AD terminology, epidemiology and risk factors, clinical phenotypes and natural history, pathophysiological mechanisms, diagnostic approaches (including imaging and fluid biomarkers), and established as well as emerging therapeutic strategies, while also outlining key challenges and future directions.

## 1. Introduction

Alzheimer's disease (AD) is the most prevalent neurodegenerative dementia, responsible for the majority of dementia cases in older adults [1]. It is distinguished clinically by an insidious onset and gradual yet relentless decline in memory and other cognitive domains [2]. Although it is often misconceived as an inevitable consequence of aging, AD is a distinct pathological entity that progressively disrupts neuronal communication, metabolism, and repair mechanisms. The disease process begins many years—often decades—before the appearance of cognitive symptoms, which complicates early recognition and limits the effectiveness of treatment initiated at the symptomatic stage [3]. As life expectancy rises globally, AD has become an escalating public health problem, prompting intense research efforts to clarify its biological underpinnings and to develop disease-modifying interventions.

A central clinical challenge lies in distinguishing the cognitive changes of normal aging from those indicative of AD. While older adults frequently report occasional word-finding difficulty, name retrieval problems, or “tip-of-the-tongue” experiences, AD is characterized by a fundamentally different pattern in which the capacity to form and retain new episodic memories progressively deteriorates [4],[5]. This decline gradually extends beyond memory, ultimately affecting multiple cognitive domains and interfering with routine activities and social or occupational roles. The course of AD can be conceptualized as a continuum spanning subjective cognitive concerns, mild cognitive impairment (MCI), and overt Alzheimer's dementia, with each step reflecting increasing neuropathological burden [6].

## 2. Terminology

Alzheimer's disease was first described in the early 20th century by Alois Alzheimer, who reported the clinical and pathological features of a young patient with presenile dementia. In 1910, Emil Kraepelin, a German psychiatrist, incorporated the condition into his influential psychiatric classification as “Alzheimer's dementia”. Notably, Kraepelin's naming largely overlooked the significant contributions of the Italian physician Gaetano Perusini, who, like Alzheimer, had made important observations on the underlying pathophysiological mechanisms. This historical detail highlights the collaborative yet sometimes underrecognized nature of scientific discovery.

The term “Alzheimer's disease” has been used variably in the literature and in clinical practice, often conflating the underlying neuropathology with its clinical manifestations. Modern frameworks recommend reserving the term “Alzheimer's disease” primarily for the underlying pathological process defined by amyloid and tau abnormalities, acknowledging that these changes may not be immediately symptomatic. Under this definition, AD encompasses both preclinical, asymptomatic biomarker-positive and clinical phases [7],[8].

Diagnostic advances now allow AD pathology to be identified in vivo without requiring brain tissue, particularly through the use of amyloid PET imaging. In this context, a patient may meet biological criteria for Alzheimer's disease based on positive amyloid and tau biomarkers even before developing recognizable memory or cognitive deficits. This shift from a purely clinical to a biomarker-based conceptualization is central to current research and therapeutic strategies [9]–[11].

## 3. Epidemiology, incidence, and risk factors

Alzheimer's disease is the most common cause of dementia, accounting for approximately 60%–80% of all dementia cases. Its prevalence is strongly dependent on age [12]. Among individuals aged 60–64 years, just over 1% are diagnosed with AD, whereas among those older than 85–90 years, the prevalence rises dramatically to roughly 20%–40% [13],[14]. Thus, while aging is the principal risk factor, it is not sufficient by itself to cause the disease; rather, age interacts with genetic, environmental, and lifestyle influences that together determine whether a pathological threshold is crossed [15].

With respect to genetic risk, the apolipoprotein E (APOE) ε4 allele is the best-characterized susceptibility factor for sporadic late-onset AD. Inheriting a single APOE ε4 allele confers an approximately threefold increase in risk, while inheriting both alleles can elevate risk by approximately eightfold [16],[17]. ApoE influences lipid handling and myelination in the brain and appears to modulate Aβ deposition and clearance [18]. In addition, rare autosomal dominant mutations in genes encoding amyloid precursor protein (APP) and the presenilin-1 (PSEN1) and presenilin-2 (PSEN2) proteins cause early-onset familial AD. These mutations shift the processing of APP toward increased production of the aggregation-prone Aβ42 peptide relative to Aβ40, thereby promoting plaque formation [19]–[21].

Genetic predisposition is further highlighted by the association between Down syndrome and early Alzheimer-type pathology. Individuals with trisomy 21 overexpress the APP gene located on chromosome 21, developing cognitive deterioration and characteristic AD neuropathology by middle age. Such patients can be viewed as a natural model of accelerated amyloid deposition and neurodegeneration [22].

Beyond genetics, numerous modifiable and environmental risk factors have been identified. Advanced age remains the strongest predictor. Female sex is associated with a higher prevalence, likely reflecting both hormonal and longevity-related factors. Vascular and lifestyle factors—including hypertension, physical inactivity, current smoking, diabetes, dyslipidemia, and untreated chronic inflammation—are all linked to elevated AD risk. Chronic inflammatory conditions such as psoriatic arthritis have been implicated in promoting sustained microglial activation and cytokine release, which can influence amyloid and tau pathology. Head trauma, particularly moderate or severe traumatic brain injury, increases the probability of later-life dementia. Family history of dementia independently adds to the risk, integrating both genetic and shared environmental influences [23],[24]. Additionally, rare cases of iatrogenic amyloid-β transmission have been described in recipients of cadaveric pituitary-derived growth hormone, paralleling observations in cerebral amyloid angiopathy and iatrogenic Creutzfeldt–Jakob disease [25],[26].

Socioeconomic and psychosocial factors modulate the age at which symptoms emerge. Individuals with higher formal education, higher income, complex occupational roles, and robust social and family support tend to exhibit greater cognitive reserve. This reserve enables compensation for early neural changes; therefore, such individuals often present clinically at a later stage despite more advanced morphologic changes in imaging. Consequently, when they eventually come to medical attention, their structural brain alterations, especially atrophic changes, may appear strikingly advanced [27].

## 4. Clinical features and disease progression

### 4.1. Typical presentation

Clinically, AD most often begins with progressive impairment of episodic memory. Patients commonly present with difficulty forming new memories, misplacing objects, repeating questions, or forgetting recent conversations, while remote memories from earlier life are relatively preserved in the initial stages [28]. As pathology spreads beyond the medial temporal lobe, deficits extend to executive and attentional processes, resulting in impaired planning, organization, and problem-solving. Language disturbances, particularly word-finding difficulty, may emerge early but are typically mild. Semantic memory and visuospatial abilities decline more gradually, becoming increasingly evident in mild dementia stages as cortical involvement broadens [29].

Neuropsychiatric symptoms are frequent and may appear early in the disease course. Apathy is particularly common and often precedes marked functional decline. Depression, anxiety, irritability, agitation, and sleep disturbances may develop as the disease progresses, and psychotic features such as delusions or hallucinations can occur in advanced stages [30]. Ultimately, progressive cognitive and behavioral impairment leads to loss of independence in instrumental and basic activities of daily living, culminating in complete dependency.

### 4.2. Atypical variants

In addition to the classical amnestic presentation, AD can underlie several atypical clinical syndromes. Posterior cortical atrophy presents predominantly with visual and visuospatial disturbances, including difficulties with spatial orientation, object or face recognition, reading, and visually guided movements [31],[32]. The logopenic variant of primary progressive aphasia is characterized by pronounced word-finding pauses and impaired repetition of phrases or sentences, in the context of relatively preserved grammar and motor speech [33]. A frontal variant of AD resembles behavioral variant frontotemporal dementia, with early changes in personality, judgment, social behavior, and executive functioning [34].

These atypical variants highlight that AD is not solely an amnestic disorder but a heterogeneous neurodegenerative process whose clinical expression depends on which cortical networks are most prominently affected. Slowly progressive focal cortical atrophy in specific regions leads to symptom clusters that were historically described as distinct disorders but are now recognized to share underlying Alzheimer-type pathology [35]–[38].

## 5. Subjective cognitive decline and mild cognitive impairment

Historically, mild cognitive impairment (MCI) was regarded as the earliest clinically detectable stage of AD. However, attention has increasingly turned to an even earlier phase known as subjective cognitive decline (SCD). SCD refers to a persistent, self-experienced decline in cognitive ability relative to a previously normal baseline in individuals who nevertheless perform within normal limits on standardized neuropsychological tests. There is no acute event to explain this perceived decline, yet the individual senses that cognition is not as sharp as before.

Importantly, SCD is defined in the absence of objective cognitive deficits measurable on conventional tests and in the absence of a diagnosis of MCI or dementia. It also excludes situations in which cognitive complaints can be fully accounted for by other neurological or psychiatric conditions, medications, or substance use [39]. SCD is of great interest as a potential target for early pharmacological or disease-modifying interventions aimed at interrupting the pathological cascade before measurable impairment appears.

MCI, by contrast, denotes objective cognitive decline that is greater than expected for age and education, without significant disruption of everyday functional abilities. The patient is neither cognitively normal nor demented. Evidence of MCI may arise from objectively documented decline over time or from a combination of subjective complaints reported by the patient or informant and measurable deficits on cognitive testing. Activities of daily life remain essentially preserved, although more complex instrumental tasks may be mildly affected or require greater effort and compensatory strategies. By the time a patient reaches the MCI stage, it may already be too late for some disease-modifying treatments to fully prevent progression, as pathological changes are well underway [40].

## 6. Pathological stages

Alzheimer's disease progression can be conceptualized through complementary anatomical, biological, and clinical staging frameworks. Traditionally, pathological staging has been based on the topographic spread of neurofibrillary tangles and amyloid plaques. Braak and Braak described the hierarchical propagation of tau pathology, beginning in the transentorhinal and entorhinal cortex (Braak stages I–II), extending to limbic regions including the hippocampus (stages III–IV), and ultimately involving widespread neocortical association areas (stages V–VI). This anatomical progression closely parallels the evolution of cognitive impairment [41]–[44].

In the earliest biologically defined stage—often referred to as preclinical AD—individuals demonstrate abnormal amyloid (A+) and often early tau (T+) biomarkers but remain cognitively intact. At this stage, amyloid deposition may already be widespread, while tau pathology is initially confined to medial temporal structures. Structural imaging may be normal or show only subtle regional volume loss. This biomarker-based conceptualization is formalized in the 2018 National Institute on Aging–Alzheimer's Association (NIA–AA) research framework, which defines Alzheimer's disease biologically using the AT(N) system [7],[8].

As tau pathology extends beyond the medial temporal lobe into association cortices, neurodegeneration (N+) becomes measurable, reflected by hippocampal atrophy, cortical thinning, and network disconnection. Clinically, this corresponds to mild cognitive impairment (MCI) due to AD, characterized by objective cognitive decline—typically in episodic memory—without complete loss of functional independence [40].

In the dementia stage, widespread neocortical involvement leads to substantial cortical atrophy, ventricular enlargement, and profound synaptic and neuronal loss. Cognitive deficits extend beyond memory to include executive, language, and visuospatial domains, ultimately resulting in severe functional dependency [42],[43].

Importantly, the biological process of Alzheimer's disease begins years to decades before clinical symptoms emerge. The AT(N) framework emphasizes that Alzheimer's disease is fundamentally defined by its biomarker profile rather than its clinical presentation, and that anatomical, molecular, and symptomatic stages are related but temporally dissociated [7],[8],[36].

## 7. Detailed pathophysiology

There is a continuum between changes associated with normal aging and those characteristic of AD. Neuropathological studies have shown that many cognitively intact elderly individuals harbor the pathological features commonly associated with AD, including amyloid plaques and neurofibrillary tangles. Such findings suggest that AD pathology can be present in the absence of clinical dementia and that additional factors—such as the extent, distribution, and interplay of these lesions with other brain processes—determine whether cognitive decline becomes apparent [44].

Alzheimer's disease disrupts three fundamental processes critical to neuronal health: communication, metabolism, and repair. In AD, these functions are compromised, leading to reduced synaptic efficacy, impaired energy homeostasis, and diminished capacity for cellular recovery. Senile plaques and neurofibrillary tangles are the most widely recognized histopathological hallmarks; however, neuronal and synaptic loss, glial activation, oxidative stress, and vascular changes all contribute to cognitive decline [45]. While amyloid plaques are believed to precede clinical onset, their sheer number and distribution correlate poorly with clinical severity. In contrast, the burden and topography of neurofibrillary tangles are strongly associated with the degree of cognitive impairment.

### 7.1. Senile plaques

Senile plaques are dense, largely insoluble extracellular deposits composed of Aβ peptide and associated cellular debris accumulated around neurons. Aβ is derived from the amyloid precursor protein (APP), a transmembrane protein, via proteolytic cleavage. In AD, Aβ fragments aggregate, often in association with neurons, glial cells, and other molecules [46]. Plaques typically accumulate in the hippocampus—which is crucial for encoding new memories—and, in association, cortices involved in higher-order cognition and decision-making. Notably, plaque deposition may begin as early as the fifth decade of life.

Whether Aβ plaques themselves are directly neurotoxic or primarily a by-product of upstream processes remains unresolved, though the prevailing view is that amyloids play a key initiating role in the pathogenesis of AD. Mutations in APP that alter the structure or processing of the protein can cause rare inherited forms of AD, underscoring the importance of APP metabolism. Aβ accumulation occurs to a limited degree in nearly all aging brains but is markedly increased in AD, suggesting a quantitative and qualitative shift in clearance and aggregation dynamics [47],[48].

#### 7.1.1. Soluble Aβ oligomers and synaptotoxicity

Although fibrillar plaques are the most visible neuropathological hallmark of AD, extensive experimental and clinicopathological evidence suggests that soluble misfolded Aβ assemblies (oligomers) may be the most neurotoxic Aβ species and can exert deleterious effects well before extensive plaque deposition [49]. Soluble Aβ oligomers disrupt synaptic plasticity and are strongly linked to early cognitive impairment through impairment of long-term potentiation, spine loss, and altered glutamatergic signaling, including abnormal NMDA receptor–dependent calcium influx and excitotoxic stress.

Beyond direct synaptic effects, oligomeric Aβ may also amplify downstream pathology by promoting oxidative stress, impairing proteostasis, and facilitating tau-related injury (including kinase activation and phosphorylation cascades). This framework helps reconcile the relatively weak correlation between plaque burden and symptom severity and supports the concept that plaques may partly reflect a later-stage, more aggregated endpoint of earlier soluble toxicity [49],[50].

### 7.2. Neurofibrillary tangles

Neurofibrillary tangles (NT) are intracellular aggregates of hyperphosphorylated tau, a microtubule-stabilizing protein. In healthy neurons, tau binds microtubules and supports axonal transport. In AD, tau becomes aberrantly phosphorylated, dissociates from microtubules, and aggregates first into soluble oligomers and then into insoluble tangles. This process disrupts axonal transport and compromises neuronal function [51],[52].

NFTs initially accumulate in the medial and polar aspects of the temporal lobe, particularly within the entorhinal cortex and hippocampus. As the disease advances, they spread to higher-order association cortices and, less frequently, to primary sensory and motor areas. Senile plaques (SP) also primarily accumulate in association cortices and the hippocampus. Both SPs and NFTs exhibit relatively discrete and stereotyped patterns of laminar distribution in the cerebral cortex, reflecting predominant involvement of corticocortical projection neurons [53],[54].

### 7.3. Amyloid versus tau hypotheses

**Figure 1.**
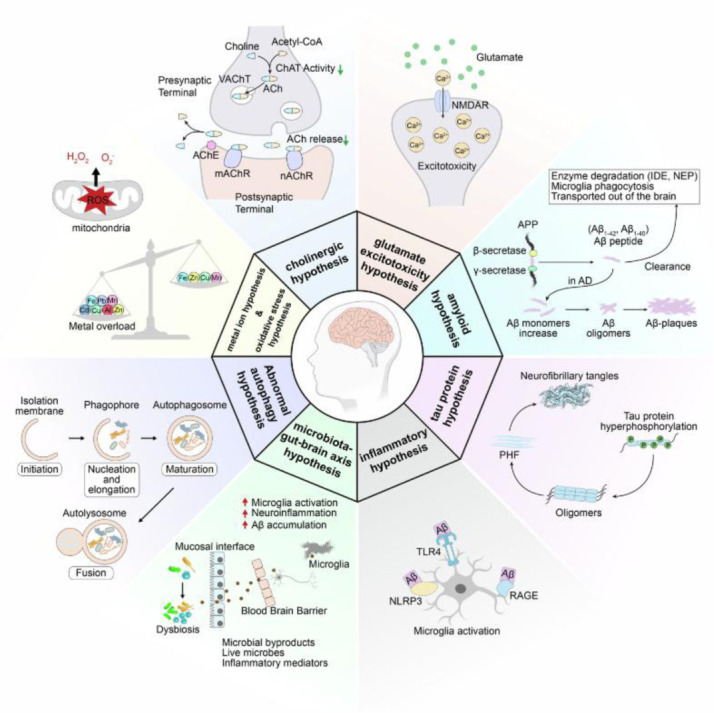
Schematic overview of Alzheimer's disease pathogenesis, encompassing the cholinergic and glutamatergic hypotheses, the amyloid and tau protein hypotheses, neuroinflammatory mechanisms, the microbiota–gut–brain axis, oxidative stress, metal ion dysregulation, and impaired autophagy [57].

Two major conceptual frameworks have been proposed to explain AD pathogenesis, though several other hypotheses have been proposed (Figure 1): the amyloid and the tau hypothesis. The amyloid hypothesis posits that elevated levels of Aβ42—a more aggregation-prone, 42–amino acid isoform of Aβ—lead to plaque formation and subsequent neuronal toxicity. Diffuse Aβ deposition is thought to precede the development of well-defined plaques and tangles. APP can be cleaved by α-, β-, and γ-secretases. In normal cellular metabolism, sequential cleavage by α- and then γ-secretase yields small, non-toxic fragments. When APP is instead cleaved by β- and then γ-secretase, Aβ40 and Aβ42 are produced. Aβ42 has a greater tendency to aggregate, and an increased Aβ42:Aβ40 ratio is considered critical for amyloid toxicity [55],[56].

The tau hypothesis emphasizes the destabilization of the microtubular system through tau hyperphosphorylation. This destabilization may impair the Golgi apparatus, alter protein processing, and possibly increase Aβ production. It also reduces axoplasmic flow, giving rise to dystrophic neurites and synaptic loss [58]. Because the distribution and burden of NFTs correlate more closely with dementia severity than plaque burden, many investigators propose that tau pathology is the chief driver of clinical decline, with amyloid serving as an upstream trigger [59].

The relative primacy of amyloid versus tau remains unresolved. What is clear is that the hippocampus and entorhinal cortex are consistently among the earliest regions affected, showing dystrophic neurites, synaptic loss, neuronal degeneration, and accumulation of both SPs and NFTs.

### 7.4. Neuroinflammation and vascular contributions as modulators of amyloid and tau pathology

Neuroinflammation is now recognized as a central component of Alzheimer's disease (AD) pathogenesis rather than a mere epiphenomenon of protein aggregation. Microglia, the resident immune cells of the central nervous system, initially play a protective role by promoting amyloid-β (Aβ) clearance through phagocytosis. However, chronic exposure to aggregated Aβ and other damage-associated molecular patterns induces sustained microglial activation, shifting these cells toward a pro-inflammatory phenotype characterized by the release of cytokines such as interleukin-1β, tumor necrosis factor-α, and interleukin-6. This persistent inflammatory state impairs Aβ clearance mechanisms and promotes further aggregation, thereby establishing a feed-forward loop.

Importantly, neuroinflammation also directly influences tau pathology. Pro-inflammatory cytokines activate intracellular kinases, including glycogen synthase kinase-3β (GSK-3β) and cyclin-dependent kinase 5 (CDK5), which enhance tau phosphorylation. In addition, activated microglia may facilitate trans-synaptic propagation of pathological tau species via extracellular vesicles and inflammatory signaling cascades. Thus, inflammation acts not only as a downstream response to amyloid deposition but also as an active modulator of tau-mediated neurodegeneration.

Vascular dysfunction represents another critical and often interacting pathway. Cerebral small vessel disease, endothelial dysfunction, and blood–brain barrier (BBB) breakdown are frequently observed in AD. Impairment of BBB integrity reduces the efficiency of Aβ efflux transporters such as low-density lipoprotein receptor-related protein 1 (LRP1) while increasing influx through receptors such as RAGE, thereby favoring cerebral amyloid accumulation. Chronic cerebral hypoperfusion and microvascular injury contribute to oxidative stress, mitochondrial dysfunction, and white matter damage, all of which exacerbate neuronal vulnerability.

The relationship between vascular pathology and classic AD proteinopathies appears bidirectional. Amyloid deposition in vessel walls, as seen in cerebral amyloid angiopathy, further compromises vascular integrity, increasing the risk of microhemorrhages and inflammatory responses. Conversely, vascular risk factors such as hypertension, diabetes, and dyslipidemia may accelerate amyloid and tau accumulation through impaired clearance and metabolic stress.

From a therapeutic perspective, inflammatory and vascular mechanisms may function both as downstream amplifiers of amyloid and tau pathology and as partially independent drivers of neurodegeneration [60]. This distinction has important implications. If neuroinflammation and vascular dysfunction merely reflect secondary responses, then amyloid- or tau-targeted therapies may suffice. However, if they represent semi-autonomous pathological processes that sustain degeneration even after amyloid plateau, then targeting microglial activation, endothelial integrity, metabolic dysfunction, and BBB restoration becomes a rational complementary strategy. Increasingly, clinical trials are investigating anti-inflammatory agents, modulators of innate immunity, and therapies aimed at improving vascular health as potential disease-modifying approaches.

### 7.5. Cholinergic neurotransmission

The cholinergic system plays a key role in memory and cognition and is notably compromised in AD. Activity of choline acetyltransferase, the enzyme responsible for synthesizing acetylcholine, and acetylcholinesterase, the enzyme that breaks it down, is significantly reduced in the cerebral cortex, hippocampus, and amygdala of individuals with AD [61]. Neurons in the basal forebrain—particularly the nucleus basalis of Meynert and the diagonal band of Broca—provide the major cholinergic input to the neocortex, hippocampus, and amygdala. These neuronal populations undergo substantial degeneration in AD [62].

Although the basal forebrain was not initially considered a primary focus of AD pathology, repeated studies have demonstrated substantial neuronal loss in this region [63]. Given the importance of cholinergic transmission for cortical function, the loss of these neurons contributes to both cognitive and behavioral symptoms. Enhancing cholinergic neurotransmission, primarily by inhibiting acetylcholinesterase and prolonging the action of acetylcholine at synapses, forms the pharmacological basis of symptomatic treatment in early and moderate stages of the disease.

### 7.6. Genetics

Most cases of AD are sporadic, but familial clustering is recognized. Autosomal dominant AD represents less than 5% of all cases and is almost always of early onset. Even among familial cases, genetic heterogeneity is substantial [64]. Mutations in APP (on chromosome 21), PSEN1 (on chromosome 14), and PSEN2 (on chromosome 1) are known to cause early-onset autosomal dominant AD, all favoring increased production or reduced clearance of Aβ42 relative to Aβ40 [65].

The discovery that individuals with Down syndrome develop AD pathology by midlife led to the identification of the APP gene on chromosome 21. Recent prospective biomarker studies in Down syndrome cohorts demonstrate a highly predictable sequence of amyloid accumulation, tau propagation, and clinical decline, reinforcing Down syndrome–associated AD as a genetically determined form of Alzheimer's disease with near-universal amyloid pathology in mid-adulthood [66]. These individuals have lifelong cognitive impairment and experience an accelerated decline in cognitive abilities compared with those without trisomy 21, making them a natural experimental model for amyloid-driven neurodegeneration [67]. Presenilins, which form the catalytic core of γ-secretase, also contribute to familial AD when mutated, further increasing relative Aβ42 levels [68].

These mutations, however, account for only a small fraction of AD cases. For late-onset AD, the *APOE* gene on chromosome 19 is a key susceptibility factor. ApoE is a lipid transport protein that binds Aβ within senile plaques and influences its clearance and deposition. The *APOE* gene is codominantly inherited and has three common alleles: ε2, ε3, and ε4. Carriage of the ε4 allele increases the risk of AD and lowers the age of onset, while ε2 may confer some protective effect [69].

Beyond increasing overall disease risk, APOE ε4 genotype also influences clinical phenotype and neuroimaging patterns. Individuals homozygous for the ε4 allele (APOE ε4/ε4) are more likely to present with a predominantly amnestic form of mild cognitive impairment and demonstrate earlier and more pronounced hippocampal atrophy on structural MRI. Moreover, APOE ε4 carriers—particularly homozygotes—often exhibit a greater burden of cerebral amyloid angiopathy, reflecting vascular amyloid deposition. This vascular component has important therapeutic implications, as APOE ε4/ε4 status is associated with increased susceptibility to amyloid-related imaging abnormalities (ARIA) during anti-amyloid monoclonal antibody treatment. Recent large-scale biomarker studies have further suggested that APOE ε4 homozygosity may represent more than a susceptibility state. Longitudinal analyses indicate that individuals with the ε4/ε4 genotype exhibit highly penetrant amyloid pathology and biomarker trajectories resembling those observed in autosomal dominant Alzheimer's disease, prompting discussion of APOE ε4/ε4 as a genetically defined biological subtype of AD [70]. While inheritance patterns differ from Mendelian mutations in APP or PSEN genes, the consistency and early onset of amyloid positivity in ε4/ε4 individuals support a reframing of this genotype as a distinct pathobiological entity within the Alzheimer's continuum.

## 8. Advances in diagnosis and biomarkers

Recent updates to diagnostic criteria further reinforce the biological definition of Alzheimer's disease. According to the 2024 revised criteria from the Alzheimer's Association Workgroup, AD is defined by the presence of core neuropathologic change, detectable through validated biomarkers, rather than solely by its clinical syndrome. Under this framework, an abnormal “Core 1” biomarker—such as amyloid PET, approved cerebrospinal fluid assays, or accurate plasma phosphorylated tau (particularly p-tau217)—is sufficient to establish a biological diagnosis of AD, even in asymptomatic individuals. “Core 2” biomarkers, including tau PET and additional biofluid measures, provide staging and prognostic information and increase confidence that AD pathology is contributing to clinical symptoms [71].

This biologically anchored definition does not replace clinical evaluation but distinguishes the underlying disease process from its symptomatic expression. Clinical staging remains essential for assessing functional impact and guiding management across the disease continuum.

Historically, AD diagnosis relied on clinical assessment supported by structural imaging and laboratory tests to exclude other causes. Confirmatory diagnosis required histological demonstration of plaques and tangles at autopsy or, rarely, brain biopsy. The development of fluid and imaging biomarkers has dramatically altered this paradigm, enabling in vivo detection of pathology and earlier diagnosis [72].

CSF assays are well-established markers of core AD pathology. Reduced CSF Aβ42 reflects its sequestration into plaques, whereas elevated total tau (t-tau) and phosphorylated tau (p-tau) indicate axonal injury and tangle formation [73]. PET imaging with amyloid and tau tracers allows visualization of these pathologies in vivo, with amyloid PET delineating cortical Aβ deposition and tau PET reflecting the distribution of NFTs. Such imaging supports staging across preclinical, MCI, and dementia phases and is increasingly used in research and selected clinical scenarios.

More recently, blood-based biomarkers have shown remarkable progress. Plasma measurements of p-tau217, p-tau181, and Aβ42/40 ratios correlate well with PET and CSF findings and have strong predictive value for progression from preclinical to symptomatic stages [74]. Because blood tests are less invasive, more accessible, and less expensive than CSF or PET, they offer substantial promise for large-scale screening and longitudinal monitoring and may ultimately transform clinical practice.

In parallel, sophisticated computational models that incorporate longitudinal structural and functional imaging data are being used to build individualized “disease trajectories”. Such models can predict future cortical atrophy, amyloid and tau spread, and cognitive decline, potentially allowing more personalized prognostication and treatment planning [75].

### 8.1. Temporal evolution of biomarkers across the Alzheimer's continuum

The progression of AD is mirrored by evolving abnormalities in various biomarkers, each becoming abnormal at a characteristic time in the disease course. These biomarker trajectories do not change linearly; rather, they tend to follow sigmoid-shaped curves. In the earliest, preclinical stage, biomarkers reflecting Aβ plaque burden become abnormal first. Cerebral amyloid deposition and corresponding cerebrospinal fluid (CSF) and positron emission tomography (PET) measures are dynamic early in the disease and often plateau by the time overt clinical symptoms emerge. For many years, Aβ-related markers were the principal biomarkers used to detect AD pathology [36].

As the disease advances into a late preclinical and prodromal phase, markers of tau-mediated neuronal injury, dysfunction, and degeneration become increasingly abnormal. These include elevated CSF tau and phosphorylated tau (p-tau), as well as tau PET signals in limbic and association cortices. Tau-associated biomarkers correlate more closely with clinical symptom severity than amyloid measures. Structural MRI changes, particularly regional atrophy, represent a later stage in the biomarker cascade. Atrophic changes, especially in medial temporal and temporoparietal regions, tend to become apparent after significant tau pathology is present, yet MRI maintains a close relationship with cognitive performance throughout later stages of the disease [37].

Large longitudinal cohort studies, particularly those from the Alzheimer's Disease Neuroimaging Initiative (ADNI), have been instrumental in refining the temporal sequence of biomarker changes. Analyses from ADNI cohorts demonstrate that hippocampal atrophy detectable on structural MRI may accelerate approximately three years prior to the onset of measurable clinical symptoms, reinforcing its role as an early marker of neurodegeneration. These findings support the concept that structural MRI changes are not merely late manifestations of established dementia but may reflect an evolving neurodegenerative process during the prodromal phase [76]. Recent comprehensive reviews further emphasize hippocampal atrophy as a robust and reproducible imaging biomarker with strong prognostic relevance across the Alzheimer's continuum [77]–[79].

Clinical manifestations follow biomarker changes. Cognitive impairment, especially in memory, becomes measurable at the MCI stage, while functional decline sufficient to impair independence defines the dementia phase. Given that the underlying biological process is often fully established by the time a clinical diagnosis is made, there is a substantial mismatch between the timing of pathology and symptoms. This temporal gap presents a major obstacle to effective treatment, and by the time cognitive deficits are recognized, neuropathological damage may already be advanced [38].

### 8.2. Biomarker-based diagnosis

Biomarker-based diagnostic criteria classify Alzheimer's disease biologically, focusing on the presence of amyloid and tau abnormalities rather than solely on the clinical syndrome. Amyloid PET typically shows cortical radiotracer uptake with loss of gray–white differentiation when Aβ deposition is present. Tau PET tracers highlight neurofibrillary pathology in regions corresponding to Braak stages and correlate strongly with the severity of cognitive impairment [69],[70]. CSF assays of decreased Aβ42 and increased tau species remain robust indicators of AD pathology [73]. Plasma biomarkers are now emerging as powerful adjuncts or alternatives, offering a feasible pathway to large-scale early detection.

### 8.3. Limitations and practical challenges of biomarker implementation

Despite remarkable advances in fluid and imaging biomarkers for Alzheimer's disease (AD), several important limitations constrain their widespread clinical implementation.

One major challenge concerns specificity. While amyloid and tau biomarkers are sensitive indicators of Alzheimer-type pathology, amyloid deposition is not synonymous with clinical dementia. A substantial proportion of cognitively normal older adults demonstrate positive amyloid PET scans or reduced CSF Aβ42 levels without manifest cognitive impairment. Similarly, elevated plasma phosphorylated tau species may be observed in individuals with other neurodegenerative or systemic conditions. Thus, biomarker positivity must be interpreted within a broader clinical and cognitive context to avoid overdiagnosis or inappropriate therapeutic intervention.

Standardization represents another critical barrier. Variability exists across assay platforms, laboratory methodologies, and reference ranges, particularly for plasma-based biomarkers. Differences in pre-analytical handling, storage conditions, and analytical techniques can significantly influence measured concentrations of Aβ and tau species. Although international efforts are underway to harmonize assays and establish universally accepted cutoffs, cross-platform reproducibility remains imperfect. Imaging biomarkers face similar issues, including inter-scanner variability, tracer differences, and reader-dependent interpretation.

Accessibility and cost further limit clinical translation. Amyloid and tau PET imaging remain expensive and are not uniformly reimbursed across healthcare systems. In many regions, PET imaging infrastructure is limited, particularly in low- and middle-income countries. Cerebrospinal fluid analysis requires lumbar puncture, which, although generally safe, may be perceived as invasive and is not always readily available in primary care settings. Blood-based biomarkers offer the greatest promise for scalable screening; however, regulatory approval, reimbursement pathways, and integration into routine workflows are still evolving.

Another unresolved issue concerns the appropriate clinical use of biomarkers in asymptomatic individuals. Identifying preclinical AD raises ethical and counseling considerations, particularly in the absence of universally accessible and highly effective disease-modifying therapies. Disclosure practices, psychological impact, and long-term monitoring strategies remain areas of active debate.

Finally, comorbid pathologies—including vascular disease, Lewy body pathology, TDP-43 proteinopathy, and mixed dementia—can complicate interpretation. Biomarker profiles may not fully capture the multifactorial nature of cognitive impairment in older adults, and reliance on single-pathology frameworks may oversimplify real-world clinical complexity.

Collectively, these limitations underscore that while fluid and imaging biomarkers represent transformative tools for research and increasingly for clinical practice, their optimal integration requires continued standardization, cost reduction, equitable access, and careful clinical contextualization.

### 8.4. Clinical diagnosis

Despite these advances, clinical evaluation remains fundamental. A clinical diagnosis integrates history from the patient and informants, neurological and mental status examination, and formal neuropsychological testing to document progressive decline in memory or other cognitive domains. Historically, the NINCDS–ADRDA criteria divided patients into definite, probable, and possible AD [80]. Definite AD required both a compatible clinical picture and histological confirmation of Alzheimer's pathology. Probable AD referred to a typical clinical syndrome without histological confirmation and demonstrated a sensitivity of about 81% and a specificity of approximately 73% for AD. Possible AD described atypical clinical presentations without alternative diagnoses, but again without histological proof. While longitudinal clinical criteria remain highly sensitive for diagnosing “a dementia” (over 90%), their specificity for AD specifically is lower, often under 70%. Furthermore, older criteria included imaging and laboratory studies primarily to exclude other causes rather than as direct evidence for AD.

**Figure 2.**
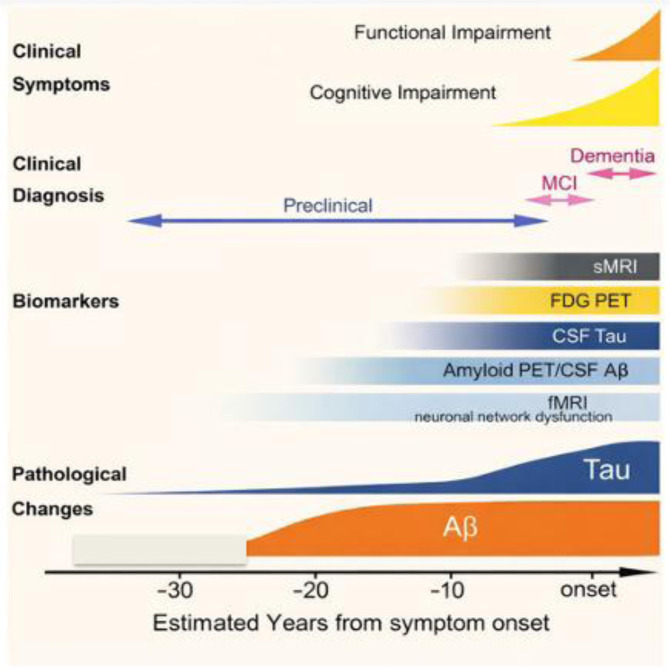
Alzheimer's disease continuum, illustrating the associated pathological processes, biomarker alterations, and stages of clinical diagnosis. Adapted from van Oostveen et al [81].

Before reliable biomarkers became available, brain biopsy was the only definitive in vivo diagnostic test, though it was rarely performed [72]. In practice, clinicians combined clinical features with structural imaging to make a probable diagnosis. The advent of CSF, PET, and blood biomarkers has now allowed much greater specificity for AD without reliance on tissue sampling (Figure 2).

## 9. Radiological and nuclear imaging features

### 9.1. Structural imaging (CT and MRI)

Although both CT and MRI can detect cortical atrophy, MRI is more sensitive and better suited for characterizing regional volume loss and excluding alternative pathologies such as multi-infarct dementia, tumors, or hydrocephalus. Structural imaging plays a critical role in AD diagnosis by identifying specific patterns of volume change.

**Figure 3.**
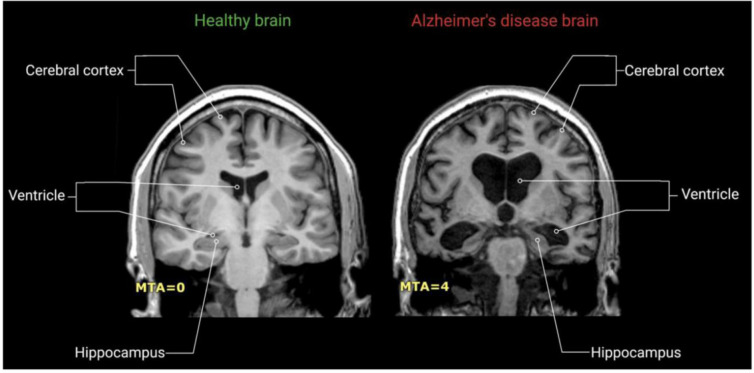
Alzheimer's disease (AD) is characterized by hippocampal atrophy and enlargement of cerebral ventricles. Compared with a healthy brain (left), the AD brain (right) shows reduced hippocampal volume, cortical thinning, and ventricular dilation. MTA is graded from 0, indicating no atrophy of the medial temporal lobe, to 4, indicating severe hippocampal volume loss.

The key diagnostic features on MRI are medial temporal lobe atrophy (MTA)—particularly involving the hippocampus, entorhinal cortex, and perirhinal cortex—and temporoparietal cortical atrophy. MTA can be assessed directly by measuring hippocampal and parahippocampal volumes or indirectly by observing enlargement of the parahippocampal fissures [82]. Direct volumetric assessment is more sensitive and specific but ideally requires quantitative techniques rather than purely visual inspection. Composite scores such as the MTA score have been developed and shown to predict progression from MCI to dementia. The entorhinal cortical atrophy score (ERICA) is another approach focusing on early atrophy in the entorhinal region (Figure 3) [83].

In patients with posterior cortical atrophy or early-onset AD, parietal and precuneus atrophy is often particularly prominent. These changes may be best appreciated along the interhemispheric surface of the parietal lobe, where enlargement of the posterior cingulate and parieto-occipital sulci and thinning of the precuneus and parietal cortex can be striking [84],[85]. Such patterns have been incorporated into scoring systems such as the posterior atrophy score (Koedam scale) [86].

While visual rating scales such as the MTA, ERICA, and Koedam scores are practical and widely used in clinical settings, they remain semi-quantitative and subject to inter-rater variability. In recent years, automated volumetric analysis tools have been increasingly incorporated into clinical and research workflows. Software platforms based on atlas-based segmentation and machine learning algorithms enable quantitative measurement of hippocampal, cortical, and whole-brain volumes, often normalized to age- and sex-matched reference databases. These approaches provide objective assessments of regional atrophy, allow detection of subtle longitudinal changes, and improve reproducibility across centers. More recently, artificial intelligence–driven tools incorporating deep learning have demonstrated high accuracy in automated brain segmentation and classification of Alzheimer's disease patterns, offering scalable and standardized evaluation of neurodegeneration. Although integration into routine clinical practice varies across healthcare systems, quantitative volumetric analysis represents an important complement to visual assessment, particularly for early detection, longitudinal monitoring, and therapeutic trials.

Quantitative studies of brain volume show that individuals with AD experience accelerated atrophy, with whole-brain volume loss averaging about 1% per year—roughly double the loss seen in normal aging (~0.5% per year). Hippocampal atrophy is even more pronounced, with AD patients losing around 4.5% of hippocampal volume annually compared with about 1.5% in healthy controls. In addition, white matter hyperintensities on T2-weighted MRI, often attributed to chronic small vessel ischemic change, correlate with cognitive impairment and may precede its clinical onset. These lesions reflect vascular contributions that can exacerbate or mimic AD pathology. White matter hyperintensities are commonly graded using the Fazekas scale, which provides a standardized visual rating of periventricular and deep white matter lesion burden and is widely applied in both clinical and research settings [87]–[89].

### 9.2. Longitudinal volumetric and microstructural MRI metrics in disease progression

While cross-sectional structural MRI is widely used to support diagnosis, longitudinal imaging provides critical insight into the temporal dynamics of neurodegeneration and network disconnection in Alzheimer's disease (AD). Repeated volumetric assessments enable quantification of regional atrophy rates and allow identification of inflection points in disease acceleration relative to amyloid and tau biomarkers.

Long-term cohort data support a staged sequence consistent with the dynamic biomarker model. In a 30-year longitudinal study of cognitively unimpaired individuals, change points in cerebrospinal fluid Aβ and phosphorylated tau were detected approximately 15–20 years before clinical symptom onset, followed by acceleration of neurofilament light chain and white matter volume changes roughly a decade before diagnosis. Notably, volumetric alterations in whole-brain white matter and ventricular expansion preceded hippocampal atrophy, highlighting that network-level degeneration may occur earlier than traditionally appreciated. These findings reinforce the concept that neurodegeneration unfolds progressively across distributed systems rather than solely within the medial temporal lobe.

Diffusion-based MRI further complements volumetric measures by detecting microstructural alterations in white matter tracts before overt macroscopic atrophy becomes evident. Diffusion tensor imaging (DTI) metrics such as fractional anisotropy and mean diffusivity capture axonal integrity and myelin organization, providing sensitive indicators of early network disruption. High-field MRI studies of post-mortem tissue have demonstrated that degeneration of perforant path fibers—connecting the entorhinal cortex to the hippocampus—occurs in preclinical stages and correlates closely with NT burden. Importantly, reductions in perforant path fiber density may precede measurable thinning of the entorhinal cortex, suggesting that microstructural disconnection can anticipate cortical atrophy.

From a pathophysiological perspective, longitudinal volumetric and diffusion-based measures bridge the gap between molecular pathology and clinical expression. Amyloid and tau biomarkers reflect upstream protein aggregation, whereas structural and microstructural MRI indices quantify downstream network failure. Because cognitive function depends on distributed neural circuits, progressive white matter degeneration and disconnection likely mediate the translation of molecular pathology into measurable cognitive impairment.

Integrating longitudinal volumetric trajectories and diffusion metrics with amyloid- and tau-based biomarkers offers a more comprehensive model of disease progression. Molecular markers identify the presence of pathological proteins; structural and microstructural imaging define the rate, anatomical distribution, and systems-level consequences of neurodegeneration. Together, these complementary modalities enhance staging accuracy, prognostic precision, and therapeutic monitoring in both clinical and research settings [90],[91].

### 9.3. Quantitative susceptibility mapping (QSM) and iron-related pathophysiology

Quantitative susceptibility mapping (QSM) has emerged as a promising MRI-based technique capable of detecting tissue magnetic susceptibility changes with high spatial resolution, primarily reflecting iron content but also influenced by myelin, calcium, and venous oxygenation. Increasing evidence suggests that altered cerebral iron metabolism contributes to Alzheimer's disease (AD) pathophysiology through oxidative stress, mitochondrial dysfunction, and interactions with amyloid-β (Aβ) and tau proteins.

Histopathological studies have demonstrated abnormal iron accumulation within amyloid plaques and in regions vulnerable to neurofibrillary degeneration, including the hippocampus and basal ganglia. Excess iron may catalyze reactive oxygen species production via Fenton chemistry, thereby exacerbating protein misfolding, tau hyperphosphorylation, and synaptic injury. QSM enables non-invasive quantification of regional iron deposition in vivo, offering a potential window into this iron-mediated pathomechanism.

Recent imaging studies have linked increased susceptibility values to amyloid burden, tau pathology, and cognitive decline. Notably, susceptibility changes in medial temporal and neocortical regions have been shown to correlate with β-amyloid deposition measured by PET and with neuropsychological performance. Furthermore, susceptibility alterations appear to interact with genetic risk factors. In individuals across the early Alzheimer's continuum, a higher apolipoprotein E (APOE) ε4 dose has been associated with impaired blood–brain barrier (BBB) water exchange rates and increased regional iron accumulation. Reduced BBB clearance efficiency correlates with both amyloid load and susceptibility-derived iron metrics, suggesting a mechanistic link between vascular dysfunction, impaired waste clearance, and metal dyshomeostasis.

Beyond iron quantification, QSM has also been explored as a tool to assess microstructural and vascular changes, including myelin alterations and venous oxygen saturation. Because iron accumulation may precede or accompany neurodegeneration, QSM could complement amyloid- and tau-based biomarkers by capturing oxidative and vascular dimensions of disease progression. Within the AT(N) research framework, susceptibility changes may intersect with both neurodegeneration (“N”) and vascular/inflammatory pathways that modulate classical proteinopathies.

However, important limitations remain. Susceptibility signals are not entirely specific to iron and may reflect mixed contributions from demyelination, calcification, or microhemorrhages. Standardization of acquisition protocols, reconstruction algorithms, and region-of-interest analyses is still evolving, and normative reference ranges are not yet firmly established. Despite these challenges, QSM represents a biologically informative and increasingly accessible imaging modality that may enrich multimodal biomarker panels, particularly in studies investigating iron metabolism, BBB dysfunction, and oxidative stress in AD [92],[93].

### 9.4. Nuclear medicine (SPECT and PET)

Nuclear medicine techniques provide functional and molecular insights that complement structural imaging. In light of the 2024 revised diagnostic criteria, these modalities can be understood according to their distinct biological roles in diagnosis and staging of Alzheimer's disease [71].

#### 9.4.1. Amyloid PET (Core 1 biomarker)

Amyloid PET imaging is considered a Core 1 biomarker under the 2024 Alzheimer's Association criteria and is sufficient to establish the biological presence of Alzheimer's disease when positive [71]. Tracers such as carbon-11 Pittsburgh compound B (PiB) and fluorine-18-labeled agents (florbetapir, flutemetamol, florbetaben) bind preferentially to fibrillar Aβ. In AD, these scans show increased cortical uptake with loss of normal gray–white matter differentiation [94]–[97].

Importantly, amyloid PET identifies the presence of Alzheimer's neuropathologic change but does not determine clinical stage. A negative amyloid PET scan makes AD biology highly unlikely. However, approximately 20%–25% of cognitively healthy older adults demonstrate positive amyloid scans, reflecting preclinical AD rather than symptomatic disease [94]–[99]. Furthermore, the magnitude of amyloid deposition does not directly correlate with cognitive severity.

#### 9.4.2. Tau PET (Core 2 biomarker and staging tool)

Tau PET tracers such as fluorine-18 flortaucipir bind to neurofibrillary tangles, producing increased uptake in regions expected to harbor tau pathology in AD, including the hippocampus, entorhinal cortex, and temporoparietal association cortices [98],[99]. Tau PET signal correlates more strongly with cognitive impairment than amyloid PET and reflects anatomical progression consistent with Braak staging.

Under the revised criteria, tau PET functions as a Core 2 biomarker, contributing to biological staging and increasing confidence that AD pathology is responsible for observed symptoms [71]. However, tau PET is not entirely specific to AD and may show positivity in other tauopathies such as chronic traumatic encephalopathy and progressive supranuclear palsy [98],[99].

#### 9.4.3. FDG-PET and SPECT (markers of neurodegeneration)

FDG-PET typically reveals bilateral temporoparietal, precuneus, and posterior cingulate hypometabolism, usually symmetric but occasionally asymmetric in early disease [94]–[99]. The anterior cingulate cortex, basal ganglia, thalami, occipital lobes (assuming eyes are open during uptake), and cerebellum tend to be relatively spared, and the sensorimotor cortices remain preserved even in late stages.

Unlike amyloid and tau PET, FDG-PET does not directly measure core AD pathology but instead reflects downstream synaptic dysfunction and neurodegeneration. Within the AT(N) framework, it contributes to the “N” category [7],[8]. SPECT imaging may demonstrate regional hypoperfusion in similar patterns but likewise lacks disease specificity.

One of the major advantages of molecular imaging is the ability to detect pathological changes before overt symptom onset. Amyloid deposition typically precedes regional tau accumulation, which is followed by reductions in glucose metabolism. This temporal sequence mirrors the biomarker cascade and supports early biological detection and staging across the disease continuum [36],[94]–[99].

## 10. AD workup and laboratory evaluation

In the current biologically defined framework, the evaluation of suspected Alzheimer's disease serves two complementary purposes: (1) to establish or confirm the presence of AD neuropathologic change using validated biomarkers, and (2) to identify alternative or coexisting conditions that may contribute to cognitive impairment. According to the 2024 revised criteria, the biological diagnosis of AD is anchored in Core 1 biomarkers, while clinical assessment and laboratory testing remain essential for staging, differential diagnosis, and management planning [100].

## 11. Treatment and prognosis

Therapeutic strategies in Alzheimer's disease can also be conceptualized according to disease stage. Primary prevention focuses on long-term risk reduction through modification of lifestyle and vascular factors in cognitively normal individuals. Secondary prevention and early intervention aim to target pathological processes during the preclinical or prodromal phases, when biomarker changes are present but functional decline is limited. Finally, treatment of established symptomatic disease seeks to slow progression or mitigate cognitive and behavioral impairment once clinical manifestations emerge. This stage-based framework complements the distinction between disease-modifying and symptomatic therapies.

Therapeutic approaches in Alzheimer's disease can be broadly divided into two principal categories: (1) disease-modifying therapies (DMTs), which target core pathology, and (2) symptomatic treatments, which address cognitive and behavioral manifestations. In addition, several emerging or adjunctive interventions are under active investigation. This framework clarifies therapeutic intent and aligns with the evolving biological understanding of AD.

### 11.1. Established symptomatic therapies

The Alzheimer's disease drug development pipeline remains highly active, with dozens of agents targeting diverse biological mechanisms across Phase I–III trials. Comprehensive annual analyses of the AD therapeutic landscape provide detailed summaries of this rapidly evolving field [101],[102].

At present, the only widely available pharmacological treatments for Alzheimer's disease are symptomatic, targeting neurotransmitter systems rather than the root causes of pathology. Standard medical therapy includes cholinesterase inhibitors (ChEIs) such as donepezil, rivastigmine, and galantamine, and the partial NMDA receptor antagonist memantine. These agents modulate acetylcholine and glutamate neurotransmission, respectively.

Cholinergic systems involved in memory and cortical information processing are impaired early in AD because of degeneration of basal forebrain neurons and corresponding reductions in cortical cholinergic innervation. Centrally acting ChEIs inhibit the breakdown of acetylcholine at the synaptic cleft, thereby partially compensating for the loss of cholinergic neurons. Initially, ChEIs were expected to only benefit early and intermediate stages when sufficient intact synapses remained. However, clinical experience has shown that they may also provide advantages in more advanced disease, in AD with concomitant infarcts, and in dementia with Lewy bodies. The main adverse effects of ChEIs are gastrointestinal and include nausea, vomiting, diarrhea, and dizziness.

Memantine is typically reserved for moderate-to-severe stages of AD. By partially blocking NMDA-type glutamate receptors, it is thought to reduce excessive calcium influx and excitotoxic neuronal damage, although the precise mechanisms remain incompletely proven. Combination therapy with memantine and ChEIs, such as donepezil, has been shown to delay institutionalization and improve outcomes in moderate-to-severe AD; however, it shows little benefit over ChEI alone in mild-to-moderate disease. Rivastigmine is also available in a transdermal patch formulation, approved for severe AD, which enhances adherence in patients with poor tolerance or compliance to oral medications [103],[104].

### 11.2. Cognitive and non-pharmacological interventions

Alongside pharmacological advances, non-pharmacological strategies are increasingly recognized as essential complementary approaches for Alzheimer's disease (AD) prevention and risk reduction, particularly during the preclinical and early disease stages. This perspective is reflected in the 2019 World Health Organization (WHO) guidelines on reducing cognitive decline and dementia risk, which emphasize addressing modifiable risk factors through lifestyle interventions, including physical activity, nutritional optimization, reduction of tobacco and harmful alcohol use, and effective management of chronic conditions such as hypertension and diabetes [105]. Although the strength of these recommendations varies due to limitations in long-term randomized data and the underrepresentation of low- and middle-income populations, the guidelines underscore the potential impact of coordinated, multidomain interventions.

Supporting this approach, the Finnish Geriatric Intervention Study to Prevent Cognitive Impairment and Disability (FINGER) and its global expansion through the World-Wide FINGERS network demonstrate that combined interventions integrating dietary modification, structured cognitive training, physical activity, and vascular risk control can preserve cognitive function in individuals at increased risk for dementia [106],[107]. Within this multidomain framework, mental stimulation and cognitively demanding activities—such as structured cognitive training programs, brainteasers, and problem-solving tasks—have been associated with delayed cognitive decline and improvements in cognitive performance and daily functioning, although their ability to substantially slow disease progression in established AD remains debated.

Nutrition has emerged as a central modifiable factor, with growing evidence that dietary patterns rich in fruits, vegetables, whole grains, unsaturated fats, and bioactive compounds—characteristic of the Mediterranean and Mediterranean-DASH Intervention for Neurodegenerative Delay (MIND) diets—are associated with reduced neuroinflammation, improved vascular health, and more favorable cognitive outcomes. Similarly, regular physical activity is consistently associated with reduced AD risk and may exert modest benefits on disease progression through vascular, metabolic, and neurotrophic mechanisms.

Complementary non-pharmacological therapies, including music-based interventions and acupuncture, have also shown potential benefits for mood, quality of life, and cerebral perfusion, though large-scale, well-controlled clinical validation is still required. Collectively, these findings support an integrated framework in which lifestyle-based, cognitive, and supportive interventions are combined with emerging disease-modifying therapies to address Alzheimer's disease across its biological continuum [108]–[110].

### 11.3. Treatment of neuropsychiatric symptoms

Behavioral and psychological symptoms of dementia—including anxiety, agitation, depression, psychosis, and sleep disturbances—can profoundly affect quality of life and caregiver burden. A variety of behavioral strategies and pharmacologic agents can be used to alleviate these manifestations. Antidepressants, anxiolytics, antiepileptic agents (frequently used for mood-stabilizing or behavioral effects), beta-blockers, antiparkinsonian agents, and antipsychotics may be employed, typically at lower doses and with careful monitoring for adverse effects [111]. There is, however, no single psychotropic drug approved specifically for treating behavioral symptoms in AD, and the choice of agent and dosing must be individualized.

### 11.4. Disease-modifying therapies and monoclonal antibodies

Over the past several years, the focus has shifted toward disease-modifying therapies (DMTs) that aim to target core pathological processes, especially amyloid-β. Multiple monoclonal antibodies have been developed to bind aggregated or soluble Aβ species and facilitate their clearance. Some of these agents, such as lecanemab and donanemab, have shown evidence of amyloid reduction and modest slowing of cognitive decline in selected patient populations. In the CLARITY-AD Phase III trial, lecanemab demonstrated statistically significant slowing of cognitive decline in early AD over 18 months, accompanied by robust amyloid reduction [112]. The TRAILBLAZER-ALZ 2 Phase III study showed significant slowing of decline in patients with early symptomatic AD and intermediate tau burden [113]. However, their long-term efficacy and real-world benefits remain the subject of ongoing evaluation, and the magnitude of clinical improvement is still limited [114]–[117].

Importantly, amyloid-lowering monoclonal antibodies can cause amyloid-related imaging abnormalities (ARIA), which include vasogenic edema (ARIA-E) and microhemorrhages or superficial siderosis (ARIA-H). ARIA is more common in APOE ε4 carriers and typically occurs within the first months of therapy. Although often asymptomatic and radiographically monitored, ARIA may occasionally present with headache, confusion, focal deficits, or seizures. Careful MRI surveillance and patient selection are therefore essential components of disease-modifying therapy protocols [98]. Aducanumab was one of the first monoclonal antibodies to receive conditional regulatory approval. The EMERGE and ENGAGE Phase III trials demonstrated mixed results, with only one of the trials meeting its primary endpoint, leading to accelerated regulatory approval based on amyloid plaque reduction (EMERGE/ENGAGE trials) [118]. However, it was ultimately discontinued in 2024 after considerable controversy regarding its clinical benefits and risk profile [119].

Other anti-amyloid antibodies that progressed to large Phase III trials but failed to demonstrate clinical benefit—such as gantenerumab and crenezumab—have further informed trial design, dosing strategies, and patient selection in subsequent programs.

Beyond plaque-directed monoclonal antibodies, increasing attention has focused on agents targeting soluble amyloid-β oligomers, which are thought to represent the primary neurotoxic species driving synaptic dysfunction. Therapeutic strategies directed at oligomeric Aβ aim to neutralize these soluble assemblies before their aggregation into fibrillar plaques and may theoretically intervene earlier in the pathological cascade. Although clinical validation remains ongoing, the oligomer hypothesis has gained renewed interest given that amyloid-directed therapies remain the only class to demonstrate reproducible, albeit modest, clinical efficacy to date. Several investigational agents (e.g., ACU193, ALZ-801) are designed to preferentially target soluble oligomeric species [120],[121]. CT1812, a small-molecule sigma-2 receptor antagonist, represents a mechanistically distinct approach that promotes displacement of Aβ oligomers from neuronal receptors and enhances their clearance into cerebrospinal fluid, with early-phase clinical studies suggesting biological target engagement [122].

Given the strong correlation between tau pathology and cognitive decline, numerous therapeutic strategies have targeted tau aggregation, phosphorylation, and intercellular propagation. These include anti-tau monoclonal antibodies and small-molecule aggregation inhibitors [123]. However, despite compelling mechanistic rationale, multiple Phase II and III clinical trials of tau-targeted therapies have failed to demonstrate significant clinical benefit to date. These outcomes suggest that tau-directed interventions may require earlier timing, improved intracellular target engagement, or combination approaches to achieve meaningful disease modification. The repeated setbacks highlight the complexity of tau biology and the challenges of translating pathological association into therapeutic success.

Beyond antibodies, numerous small-molecule strategies are under exploration, including selective enzyme inhibitors, dual-target compounds, allosteric modulators, covalent inhibitors, and proteolysis-targeting chimeras (PROTACs) designed to promote degradation of toxic proteins [124]. Other approaches aim to modulate tau phosphorylation and aggregation, stabilize normal tau conformation, enhance autophagic clearance of misfolded proteins, or reduce neuroinflammation. Pathways such as mTOR signaling, mitochondrial function, and synaptic resilience are increasingly recognized as potential therapeutic targets.

Glucagon-like peptide-1 (GLP-1) receptor agonists such as semaglutide have been investigated as potential disease-modifying therapies based on preclinical evidence suggesting modulation of neuroinflammation and metabolic dysfunction. However, results from the large Phase III EVOKE and EVOKE+ trials did not demonstrate significant slowing of clinical progression in early symptomatic Alzheimer's disease, despite evidence of favorable biomarker changes. These findings highlight the challenges of translating metabolic and inflammatory modulation into meaningful clinical benefit, while also underscoring the importance of distinguishing biomarker effects from cognitive outcomes [125].

### 11.5. Neurosurgical and experimental approaches

Deep brain stimulation and other neurosurgical interventions are being investigated as potential therapeutic modalities in AD [126]. These approaches seek to modulate neural circuits implicated in memory and cognition, though they remain experimental and are not part of standard care.

### 11.6. Inflammation, NSAIDs, and other interventions

Given the mounting evidence of intense neuroinflammation in AD, anti-inflammatory therapies have attracted interest. Epidemiological observations suggested that long-term use of nonsteroidal anti-inflammatory drugs (NSAIDs), such as aspirin, might reduce the risk of developing AD or dementia [127]. However, subsequent studies have not consistently replicated these protective effects, and the balance of evidence does not support widespread NSAID use for AD prevention. While targeting microglial pathways remains biologically compelling, not all programs have demonstrated clinical efficacy. For example, Alector's TREM2-activating antibody (AL002) failed to meet primary endpoints in a large Phase II trial, underscoring the challenges of modulating neuroinflammatory pathways in established Alzheimer's disease [128]. Nonetheless, inflammation remains a key focus of therapeutic research.

### 11.7. Natural history of treatment use

In practice, pharmacologic treatment is tailored to the disease stage. Cholinesterase inhibitors are typically initiated in early to intermediate stages of symptomatic disease and are sometimes continued into advanced stages if tolerated and beneficial [129]. Memantine, an N-Methyl-D-aspartate (NMDA) receptor antagonist, is usually introduced in intermediate to late stages and continued as long as meaningful clinical benefit is observed [130]. Some clinicians discontinue these medications in very advanced stages, such as when patients are in nursing homes with severe dementia, focusing instead on comfort-oriented care.

### 11.8. Novel treatment approaches

Traditional treatments, including cholinesterase inhibitors and NMDA receptor antagonists, provide modest symptomatic benefit by enhancing neurotransmitter signaling but do not address the underlying pathological processes driving neuronal loss and damage. In contrast, the recent approvals of monoclonal antibodies such as lecanemab (2023) and donanemab (2024) represent the first therapies shown to slow cognitive and functional decline by directly targeting amyloid-β pathology in early-stage AD. While these amyloid-directed approaches mark a significant milestone, they account for only a minority of the current therapeutic landscape. Approximately 70% of ongoing pharmacological research now targets non-canonical mechanisms linked to the cellular phase of AD. These include strategies aimed at modulating chronic microglial activation, restoring blood–brain barrier integrity, and improving cerebrovascular and metabolic resilience, reflecting recognition that inflammatory and vascular pathways may independently sustain neurodegeneration. Emerging strategies aim to regulate maladaptive microglial activation, stabilize synaptic integrity despite ongoing pathological stress, and support neuronal resilience through metabolic interventions. Notably, experimental studies demonstrating reversal of neuroinflammation, DNA damage, and cognitive deficits through restoration of cellular energy balance in animal models have challenged the long-held assumption that advanced AD pathology is irreversible, reinforcing the shift toward earlier, biologically informed, and mechanism-based therapeutic approaches.

Given the rapidly evolving landscape of Alzheimer's therapeutics, Table 1 provides a representative overview of selected disease-modifying strategies across major biological domains rather than an exhaustive catalog of all completed and ongoing clinical trials. Agents were included to illustrate key mechanistic classes, regulatory milestones, and translational challenges, including both approved therapies and selected late-stage investigational compounds. For a comprehensive and continuously updated overview of the global AD drug development pipeline, readers are referred to the annual analyses by Cummings and colleagues [101],[102].

**Table 1.** Overview of symptomatic and disease-modifying pharmacological therapies for Alzheimer's disease, categorized by therapeutic goal, AT(N) biological domain, therapeutic class, representative agents, and current clinical status. Symptomatic therapies address cognitive, psychiatric, or sleep-related manifestations of dementia and are therefore not mapped to the AT(N) framework. Disease-modifying therapies (DMTs) are organized according to their primary biological target—amyloid (A), tau (T), or neurodegeneration/non-canonical processes (N)—consistent with the NIA–AA research framework. Agents with completed or unsuccessful late-phase trials are included to illustrate key translational challenges and shifts in therapeutic strategy.

Therapeutic goal	AT(N) domain	Therapeutic class and agents	Current status	References
Symptomatic (cognitive)	-	Cholinesterase inhibitors (ChEIs): Donepezil, Rivastigmine, Galantamine	FDA approved (1996, 2000, 2001)	[131]
Symptomatic (cognitive)	-	NMDA receptor antagonist: Memantine	FDA approved (2003)	[132]
Symptomatic (psychiatric/BPSD)	-	Atypical antipsychotic: Brexpiprazole (for agitation)	FDA approved (2023)	[133]
Symptomatic (psychiatric/BPSD)	-	Dual orexin antagonist: Suvorexant (for insomnia)	FDA approved (2020)	[134]
DMT	A	Monoclonal antibody (anti-amyloid): Lecanemab	FDA approved (2023)	[114],[116]
DMT	A	Monoclonal antibody (anti-amyloid): Donanemab	FDA approved (2024)	[115]
DMT	A	Monoclonal antibody (anti-amyloid): Aducanumab	FDA accelerated approval (withdrawn 2024)	[135]
DMT	A	Monoclonal antibody (anti-amyloid): Remternetug	Phase-III trials	[136]
DMT	A	Monoclonal antibody (anti-amyloid): Solanezumab	Phase-III trials (negative)	[137]
DMT	T	Antisense oligonucleotide: BIIB080	Phase-II trials	[138]
DMT	T	Monoclonal antibody (Anti-Tau): E2814	Phase-II/III trials	[139]
DMT	N (inflammation)	GLP-1 receptor agonist: Semaglutide	Phase-III trials	[140]
DMT	N (inflammation)	Small molecule (NF-κB/ERK pathway): NE3107	Phase-III trials	[141]
DMT	N (inflammation)	Tyrosine kinase inhibitor: Masitinib	Phase-III trials	[142]
DMT	N (synaptic)	Filamin A Binder: Simufilam	Phase-III trials	[143]
DMT	N (synaptic)	HGF/MET receptor activator: Fosgonimeton	Phase-II trials	[144]
DMT	N (synaptic)	SV2A inhibitor: AGB101	Phase-II/III trials	[145]
DMT	N (genetics/precision)	Gene therapy (APOE2 delivery): LX1001	Phase I/II	[146]

Note: Abbreviations: BPSD, behavioral and psychiatric symptoms of dementia; DMT, disease-modifying therapy; FDA, U.S. Food and Drug Administration; GLP-1, glucagon-like peptide-1; NMDA, N-methyl-D-aspartate; HGF, hepatocyte growth factor; MET, mesenchymal–epithelial transition factor; SV2A, synaptic vesicle glycoprotein 2A; APOE2, apolipoprotein E ε2 isoform; NF-κB, nuclear factor kappa-light-chain-enhancer of activated B cells; ERK, extracellular signal-regulated kinase.

## 12. Differential diagnosis

The differential diagnosis of Alzheimer's disease encompasses a variety of neurodegenerative and vascular conditions that can present with similar amnestic and cognitive features. One increasingly recognized entity is limbic-predominant age-related TDP-43 encephalopathy (LATE). LATE can present with an Alzheimer-like amnestic dementia, particularly in the oldest-old, yet it is pathologically distinct. Imaging in LATE often shows severe and sometimes asymmetric volume loss in the amygdala and hippocampus with relatively sparing of other brain regions. A characteristic rostrocaudal gradient may be seen, with the amygdala affected first, followed by the hippocampal head and anterior body. Amyloid and tau PET scans are typically negative in LATE, distinguishing it from pure AD, though mixed pathologies can occur.

Other conditions with overlapping clinicoradiological features include primary age-related tauopathy (PART) and argyrophilic grain disease (AGD), which also involve tau pathology but differ in distribution and severity. Dementia with Lewy bodies represents another key differential diagnosis. It can occur independently or in conjunction with AD pathology and may also show positive amyloid PET imaging. Clinically, it often presents with fluctuating cognition, visual hallucinations, REM sleep behavior disorder, and parkinsonism, features that can assist in differentiation from typical AD [147]–[150].

In cases where bilateral mesial temporal lobe volume loss is present in an elderly amnestic patient, careful consideration of these alternative or coexisting conditions is essential. Definitive distinction among these entities often remains possible only at autopsy, although multimodal imaging and biomarker profiles increasingly aid in ante-mortem characterization.

## 13. Challenges, heterogeneity, and future directions

Despite significant advances in understanding and treating Alzheimer's disease, formidable challenges remain. One major difficulty is the marked heterogeneity of disease trajectories. Some individuals with substantial amyloid burden never develop dementia, whereas others decline rapidly despite seemingly modest pathology. This variability stems from complex interactions among genetic background, vascular and metabolic health, immune responses, cognitive reserve, and brain network architecture. Such heterogeneity complicates clinical trial design and underscores the limitations of one-size-fits-all therapeutic strategies.

The failure of many past trials targeting amyloid or tau alone highlights the importance of timing and the need for multi-target approaches. Interventions may need to be introduced during the preclinical or very early symptomatic phases to meaningfully alter disease course, before extensive neuronal loss has occurred. Achieving this goal requires robust, affordable, and scalable biomarker tools—such as plasma assays and automated imaging metrics—to detect AD pathology in asymptomatic individuals. In addition, more precise stratification of patients according to biomarker profiles, genetics, comorbidities, and network vulnerability will be crucial to tailor therapies and maximize benefits.

Given the multifactorial nature of AD, it is increasingly recognized that targeting a single pathway is unlikely to produce dramatic results. Combinatorial strategies that simultaneously reduce amyloid burden, modulate tau pathology, attenuate neuroinflammation, protect synapses, and optimize vascular and metabolic health may be more effective. Progress toward this vision will depend on advances across many disciplines, including molecular and systems neuroscience, pharmacology, imaging, computational modeling, and public health.

Preventive approaches deserve particular emphasis. Because vascular risks, metabolic syndrome, physical inactivity, and unhealthy dietary patterns significantly modulate AD risk and expression, public health initiatives that promote cardiovascular health, physical exercise, balanced nutrition, and cognitive and social engagement have the potential to reduce the incidence or delay the onset of dementia at a population level.

## 14. Conclusions

Alzheimer's disease remains one of the most formidable challenges in modern medicine. Once regarded mainly as a disorder defined by the presence of amyloid plaques and tau tangles in individuals with dementia, it is now understood as a biologically defined, multifactorial disease that begins decades before symptom onset. Protein misfolding, neuroinflammation, vascular and metabolic disturbances, synaptic failure, and large-scale network disruption all contribute to its complex pathophysiology.

The long preclinical phase of AD offers a window for early detection and intervention; however, capturing and effectively treating individuals in this phase demands refined biomarker strategies, individualized risk stratification, and safe, well-tolerated disease-modifying agents. The advent of anti-amyloid monoclonal antibodies, the rapid development of blood-based biomarkers, and growing evidence supporting lifestyle interventions represent important milestones. However, translating biomarker advances into routine global clinical practice will require continued progress in assay harmonization, cost reduction, regulatory alignment, and equitable healthcare access.

The future of Alzheimer's disease management will likely rest on integrative, multimodal strategies that combine pharmacological therapies, lifestyle and preventive measures, and personalized biomarker-guided approaches. The ultimate goal is not merely to alleviate symptoms but to delay, attenuate, or perhaps one day prevent the onset of clinical dementia altogether.

## Use of AI tools declaration

The authors declare they have not used Artificial Intelligence (AI) tools in the creation of this article.
